# Common promoter variants of the *NDUFV2 *gene do not confer susceptibility to schizophrenia in Han Chinese

**DOI:** 10.1186/1744-9081-6-75

**Published:** 2010-12-29

**Authors:** Wen Zhang, Xiaogang Chen, Wei Gong, Jinsong Tang, Liwen Tan, Hao Guo, Yong-Gang Yao

**Affiliations:** 1 Key Laboratory of Animal Models and Human Disease Mechanisms of the Chinese Academy of Sciences & Yunnan Province, Kunming Institute of Zoology, Kunming, Yunnan 650223, China; 2 The Institute of Mental Health, the Second Xiangya Hospital, Central South University, Changsha, Hunan 410011, China; 3 Graduate School of the Chinese Academy of Sciences, Beijing 100039, China; 4 School of Life Sciences, University of Science and Technology of China, Hefei, Anhui 230027, China; 5 Department of Cardiology, Calmette Hospital, Kunming Medical College, Kunming, Yunnan 650011, China

## Abstract

**Background:**

The NADH-ubiquinone oxidoreductase flavoprotein gene (*NDUFV2*), which encodes a 24 kD mitochondrial complex I subunit, has been reported to be positively associated with schizophrenia and bipolar disorder in different populations.

**Methods:**

We genotyped the promoter variants of this gene (rs6506640 and rs1156044) by direct sequencing in 529 unrelated Han Chinese schizophrenia patients and 505 matched controls. Fisher's Exact test was performed to assess whether these two reported single nucleotide polymorphisms (SNPs) confer susceptibility to schizophrenia in Chinese.

**Results:**

Allele, genotype and haplotype comparison between the case and control groups showed no statistical significance, suggesting no association between the *NDUFV2 *gene promoter variants and schizophrenia in Han Chinese.

**Conclusion:**

The role of NDUFV2 played in schizophrenia needs to be further studied. Different racial background and/or population substructure might account for the inconsistent results between studies.

## Background

Mitochondrial dysfunction was considered as a risk factor for the onset of schizophrenia and other psychiatric disorders [1]. As a core component of mitochondrial respiratory chain, the 24 kD subunit of mitochondrial complex I, NADH-ubiquinone oxidoreductase flavoprotein (NDUFV2), is a hot candidate target for psychiatric disorders [2-7]. The first clue that indicated a potential positive association of the *NDUFV2 *gene with schizophrenia could be traced to the pioneer association and linkage study by Schwab and colleagues [8], in which they reported chromosome 18p (which contains this gene), conferred susceptibility to functional psychoses in families with schizophrenia. Subsequent analyses showed a decreased level of *NDUFV2 *expression in postmortem prefrontal cortex and striatum of schizophrenia patients [9-11] and in lymphoblastoid cell line of Caucasian schizophrenia patients [4], suggesting an active involvement of NDUFV2 in schizophrenia. Recent association studies further indicated that the *NDUFV2 *promoter haplotype, which was constituted by two SNPs (rs6506640-rs1156044), was significantly associated with schizophrenia in Japanese population [5]. This association was also found in patients with bipolar disorder from different populations [6,7,12,13]. Among these studies, SNP rs1156044 (which is located in position -602 relative to the transcription start site) was suggested to be responsible for the altered expression change in patients. In particular, the "A" allele of this SNP could lead to a significantly reduce of the promoter activity [12]. Note that this seemingly deleterious A allele is the major allele in the HapMap populations [14]. Assume that the altered expression of the *NDUFV2 *gene account for the reported positive association, one would expect more people suffer from this "abnormal" allele. In this study, we genotyped two promoter variants (rs6506640 and rs1156044) of the *NDUFV2 *gene in a case-control cohort to assess their effect on schizophrenia susceptibility in Han Chinese.

## Methods

### Subjects

A total of 529 unrelated patients with schizophrenia and 505 matched healthy controls, all of Han Chinese origin, were recruited from Hunan Province in South Central China. The schizophrenia was clinically diagnosed according to DSM-IV. Informed consent was obtained from all participants or the supervisors of the patients prior to this study. Diagnosis and review of psychiatric case records were independently checked and verified by two senior psychiatrists. The controls were clinically diagnosed as no psychiatric disorders or other diseases and were well matched in geographic origin and ethnicity with the schizophrenia patients. The institutional review boards of Kunming Institute of Zoology and Central South University approved this study.

### Genotyping

The two SNPs (rs6506640 and rs1156044) were amplified independently by PCR and were genotyped by direct sequencing method using ABI PRISM 3730 Genetic Analyzer (Perkin-Elmer Applied Biosystems). PCR amplification was performed in a volume of 25 μL containing primer pair for each SNP (for rs6506440: 5'-AAAGACGGTGGTCTACTGTG-3'/5'-GGTCTCCCAACCCTAATC-3'; for rs1156044: 5'-CAGAAAAGAAGGCAGTGACG-3'/5'-CCTCATGGAGAGCCTTGTG-3'). The PCR primers were also used for sequencing. Sequencing results were handled by the DNASTAR program (DNASTAR Inc.) and the original sequencing chromatograms of each sample were further checked by eyes. A few samples were randomly selected and were independently sequenced to evaluate the genotype accuracy, and the results were well matched between different runs.

### Statistical analysis

The allele, genotype and haplotype frequencies of both SNPs were compared between case and control samples by the Fisher's Exact Probability Test. Deviation from the Hardy-Weinberg equilibrium was calculated by using Monte Carlo permutation test through HWsim program [15]. Linkage disequilibrium (LD) was calculated by Arlequin3.01 software [16]. PHASE2.1.1 program [17] based on the Bayesian method was used to infer haplotype of the two SNPs.

## Results and discussion

No deviation from the Hardy-Weinberg equilibrium was found for both rs6506640 and rs1156044 in the control subjects and schizophrenia patients in our study (Table 1). The two SNPs were strongly linked with each other (|*D'*| = 0.98, r^2 ^= 0.95 in controls; |*D'*| = 0.98, r^2 ^= 0.93 in patients). Consistent with previous reports in Japanese [5], we observed no association in allele and genotype comparisons between the case and control groups (Table 1). There was no significant difference in haplotype frequencies (Table 2) between the case and control groups; these results were in contrast to previous finding for positive associations between haplotypes A-G, G-A and G-G with schizophrenia [5].

**Table 1:** Distribution of the *NDUFV2* gene promoter variants in Han Chinese with and without schizophrenia

SNP and population	Genotype			*P*-value^a^	Allele		*P*-value^a^	HWE^b^
					
	A/A (%)	A/G (%)	G/G (%)		A (%)	G (%)		
rs6506640								
SZ-HC (n = 529)	294 (55.6)	196 (37.0)	39 (7.4)	0.179	784 (74.1)	274 (25.9)	0.113	0.551
CT-HC (n = 505)	298 (59.0)	183 (36.2)	24 (4.8)		779 (77.1)	231 (22.9)		0.689
rs1156044								
SZ-HC (n = 529)	292 (55.2)	195 (36.9)	42 (7.9)	0.142	779 (73.6)	279 (26.4)	0.075	0.396
CT-HC (n = 505)	299 (59.2)	180 (35.6)	26 (5.2)		778 (77.0)	232 (23.0)		0.914

Note: SZ = schizophrenia; HC = Han Chinese; CT = control

^a ^Two-tailed Fisher's Exact Probability Test was used to quantify the allele difference between the case and control groups

^b ^The Hardy-Weinberg Equilibrium was computed by Monte Carlo permutation test (10000 simulations)

**Table 2:** Haplotype distributions of SNPs rs6506640-rs1156044 in Han Chinese with and without schizophrenia

Haplotype	A-A (%)	A-G (%)	G-A (%)	G-G (%)	*P*-value^a^
SZ-HC	774 (73.2)	10 (0.95)	5 (0.47)	269 (25.4)	0.232
CT-HC	774 (76.6)	5 (0.5)	4 (0.4)	227 (22.5)	

Note: SZ = schizophrenia; HC = Han Chinese; CT = control

^a ^two-tailed Fisher's Exact Probability Test *P *value

Compared with western Eurasian, Chinese population is genetically close to Japanese population and they may share nearly same genetic background in disease. In order to unravel the reason of non-replicated association between *NDUFV2 *gene polymorphisms and schizophrenia in Han Chinese population, we downloaded the normal Han Chinese and Japanese data from HapMap and performed a comparison to discern potential bias in sampling. As only rs1156044 was available in HapMap database, we performed intra-population comparison for this SNP (Table 3). Our control population (CT-HC; sample 2 in Table 3) was significantly different from the Han Chinese data in HapMap (CT-CHB+CHD; sample 5) in allele frequency (*P *= 0.03) but not for genotype frequencies (*P *= 0.076). In contrast, the Japanese control population (CT-JP; sample 4) reported by Washizuka et al. [5] was not significantly different to the HapMap Japanese data (CT-JPT; sample 6) in allele frequency (*P *= 0.663), but reached statistical difference in genotype frequency (*P *= 0.041). The difference between Han Chinese control populations reported in this study and HapMap was caused by CT-CHB (CT-HC vs. CT-CHB, *P *= 0.032; CT-HC vs. CHD, *P *= 0.251), as this population was from north China and populations from north and south China are quite different according to a recent genome-wide assay [18]. All the subjects recruited in this study were from Hunan Province which is located in southern China, thereby presented genetic difference to CT-CHB. The exact reason for the observed difference between the Japanese control population and those from HapMap was unknown, as there was no detailed information regarding the Japanese control sample in Washizuka et al.'s study [5]. It thus seems that potential bias on non-random sampling and population substructure might account for the marvelous differences in both allele and genotype frequencies of rs1156044 in different populations (Table 3).

**Table 3:** Analysis of the reported allele and genotype frequencies of rs1156044 in Han Chinese and Japanese

Population and sample size	Genotype			*P*-value^a^	Allele		*P*-value^a^	Data source
					
	A/A (%)	A/G (%)	G/G (%)		A (%)	G (%)		
1. SZ-HC (n = 529)	292 (55.2)	195 (36.9)	42 (7.9)	0.023 (1 vs. 3)	779 (73.6)	279 (26.4)	0.005 (1 vs. 3)	This study
				0.018 (1 vs. 4)			0.044 (1 vs. 4)	
				0.346 (1 vs. 5)			0.459 (1 vs. 5)	
2. CT-HC (n = 505)	299 (59.2)	180 (35.6)	26 (5.2)	0.0003 (2 vs. 3)	778 (77.0)	232 (23.0)	0.0004 (2 vs. 3)	This study
				0.001 (2 vs. 4)			0.001 (2 vs. 4)	
				0.076 (2 vs. 5)			0.030 (2 vs. 5)	
3. SZ-JP (n = 212)	94 (44.3)	95 (44.8)	23 (10.8)	0.264 (3 vs. 6)	283 (66.7)	141 (33.3)	1.000 (3 vs. 6)	Washizuka et al. 2006
4. CT-JP (n = 222)	99 (44.6)	106 (47.7)	17 (7.7)	0.041 (4 vs. 6)	304 (68.5)	140 (31.5)	0.663 (4 vs. 6)	Washizuka et al. 2006
5. CT-CHB+CHD (n = 243)	123 (50.6)	103 (42.4)	17 (7.0)	0.055 (5 vs. 6)	349 (71.8)	137 (28.2)	0.187 (5 vs. 6)	HapMap dataset
6. CT-JPT (n = 113)	55 (48.7)	41 (36.3)	17 (15.0)	--	--	--	--	HapMap dataset

Note: SZ-HC and CT-HC refer to Han Chinese with and without schizophrenia, respectively. CT-CHB = Beijing Han Chinese from HapMap; CHD = Chinese in Metropolitan Denver from HapMap; CT-JPT = Japanese in Tokyo from HapMap; SZ-JP and CT-JP refer to Japanese patients with and without schizophrenia from Washizuka et al. [5]. We did not include the reported Han Chinese data in Zhang et al's study [7] for comparison, as there is inconsistency about the sample size in their text. ^a ^two-tailed Fisher's Exact Probability Test *P *value

Despite a fact that "A" allele of rs1156044 was reported to decrease the promoter activity in functional assay [12], this functional deficiency does not seem to be the causal factor for schizophrenia in Han Chinese. The lack of association in allele, genotype and haplotype with schizophrenia in our study indicates that the proposed effect of the *NDUFV2 *gene on schizophrenia should be treated with caution. Racial difference and/or population substructure may account for the inconsistent results between Han Chinese and Japanese as observed in this study. It should be mentioned that the most significant haplotypes (A-G and G-A) found by Washizuka et al. [5] were rare haplotypes, which could be more easily influenced by the small sample size (199 patients vs. 221 controls). Evidently, the utilization of public HapMap database undoubtedly provides insightful information in association study, especially when inconsistent results were reported.

## Limitations

There are several limitations that should be addressed in the present study. First, we did not sequence the entire promoter region of the *NDUFV2 *gene and no other tagging SNPs of this gene was genotyped, so we could not rule out the possibility that other unreported promoter variant(s) and/or haplotype(s) affected the expression of this gene. Second, only Han Chinese sample from Hunan Province was recruited in this study, and this might not necessarily eliminate the potential bias regarding the sampling. Finally, we did not put the data for other confounding factors (e.g. age, sex and so on) into the logistic regression model to test whether they would affect the associations for those two SNPs, as we thought that the division of the current medium sized case-control samples might cause type I error.

## Conclusions

As increasing evidence to support "common disease rare variant model" in schizophrenia in recent two years [19,20] and seldom replication of common variant mainly based on genome wide association studies (GWASs) [21-33], we should be more careful to report association between common variant (e.g. rs6506640 and rs1156044, all with MAF≈0.3) and schizophrenia. More studies with large sample size and population from different regions, as well as, functional assays should be conducted to finally elucidate the role of NDUFV2 in schizophrenia.

## Competing interests

The authors declare that they have no competing interests.

## Authors' contributions

YGY, XC, WZ designed the study, XC, JT, LT are responsible for the inclusion of patients and controls. WZ, WG, HG performed the experiments and statistical analyses. WZ and YGY wrote the manuscript. All authors contributed to and approved the final version of the manuscript.
